# Meteorological Register

**Published:** 1814-03

**Authors:** 

**Affiliations:** Philosophical Instrument Maker, No. 38, Tavistock-Street, Covent-Garden


### METEOROLOGICAL REGISTER.

*From January 25, 1813, to February 25, 1814.*

Kept by Mr. BLUNT, Philosophical Instrument Maker, No. 38, Tavistock-Street, Covent-Garden.

| Moon. | Day. | Wind. | Barometrical Pressure. Max. | Barometrical Pressure. Min. | Temperature. Max. | Temperature. Min. | |
|---|---|---|---|---|---|---|---|
| | 26 | NW | 29·59 | 29·36 | 33 | 24 | Sleet |
| | 27 | W | 29·21 | 29 11 | 39 | 33 | Clear |
| | 28 | W | 29·25 | 29·10 | 39 | 25 | Rain |
| ◐ | 29 | E | 29·— | 28·22 | 38 | 24 | Clear |
| | 30 | W | 29 30 | 29·10 | 39 | 26 | Clear |
| | 31 | W | 29 41 | 29 29 | 36 | 32 | Clear |
| | 1 | N | 30 02 | 29·85 | 40 | 31 | Clear |
| | 2 | NW | 30·— | 29 88 | 34 | 24 | Clear |
| | 3 | NW | 30.01 | 30 — | 30 | 21 | Clear |
| ○ | 4 | NW | 30 18 | 30·03 | 31 | 21 | Clear |
| | 5 | SE | 30 07 | 29 88 | 37 | 25 | Cloudy |
| | 6 | W | 29 60 | 29·54 | 45 | 26 | Rain |
| | 7 | NW | 29·68 | 29 62 | 46 | 33 | FlsClouds |
| | 8 | W | 29·59 | 29 39 | 47 | 38 | Rain |
| | 9 | NW | 29·84 | 29 63 | 48 | 41 | Clear |
| | 10 | W | 30·03 | 29 01 | 49 | 42 | Fine |
| | 11 | W | 29·98 | 29·93 | 48 | 43 | Fine |
| ◑ | 12 | NW | 29 95 | 29·95 | 50 | 41 | Fine |
| | 13 | SW | 29 95 | 29.90 | 52 | 45 | Cloudy |
| | 14 | E | 30·02 | 29·89 | 38 | 33 | Fair, with flying Clouds |
| | 15 | E | 30 08 | 30 04 | 36 | 30 | Fair |
| | 16 | E | 30·22 | 30 13 | 32 | 21 | Fair |
| | 17 | NE | 30 42 | 30·36 | 39 | 26 | Fair. Rain at 3 p. m. |
| | 18 | NW | 30 35 | 30 22 | 40 | 25 | Rain |
| | 19 | W | 30 29 | 30·21 | 30 | 21 | Fair |
| ● | 20 | E | 30·40 | 30 40 | 34 | 22 | Fair |
| | 21 | NE | 30·30 | 30 26 | 31 | 24 | Fair |
| | 22 | E | 30·27 | 30·24 | 30 | 23 | Fair |
| | 23 | E | 30 23 | 30·15 | 30 | 18 | Fair |
| | 24 | E | 0·15 | 30·13 | 32 | 19 | Fair |

RESULTS.

| | | | |
|---|---|---|---|
| Mean barometrical pressure 29·837 | | Mean temperature | 33 |
| Maximum 30·42, the wind at | NE | Maximum 52, the wind at | SW |
| Minimum 28·22, ——— | E | Minimum 18, ——— | E |

*Scale exhibiting the prevailing Winds during the Month.*

| N | NE | E | SE | S | SW | W | NW |
|---|---|---|---|---|---|---|---|
| 1 | 2 | 8 | 1 | 0 | 1 | 9 | 8 |

January 26th, a thaw commenced, which continued during the six following days. Frost, at a temperature of 24 to 32, prevailed during the nights. A gradual breaking up of the ice and snow was thus effected, and the anticipated inconveniences and damages commonly produced by a sudden thaw, in a great measure, prevented. The Thames was again open to the passage of craft, and the roads to carriages.

Scenes seldom witnessed in this country have occurred during this severe and unusual frost. Secure beaten paths on the ice, formed on various parts of the river between the bridges of the metropolis, and kept in order by the watermen of the different landing places, became frequented thoroughfares.

roughfares. Many booths of entertainment were erected, and at Chiswick a sort of fair was held on the ice, in which were shews of the usual description; in one instance a sheep was roasted: amid these diversions few fatal accidents have been noticed.

Serious casualties in the streets of the metropolis have seldom occurred, but in those instances of reprehensible neglect, where the accumulations of ice have been suffered to obstruct the carriage and foot ways, after the thaw of 26th January; and these still occur in the carriage-way in many of the best streets.

Feb. 3d.—The frost again set in, and has since continued with little variation. It was now relieved from the fogs and snow which characterised the first part of it. The thermometrical measure of its intensity is nearly similar.

During the thaws which took place, pieces of still water, or those not influenced by rapid streams, still continued to afford the amusement of skaiting to the adventurers. In such places, fish may at this time be seen frozen, in their natural position, in the solid ice.

An unusual privation of water is still suffered in the metropolis.

Looking to the effect of this frost, it may certainly be considered to have existed from the commencement in December, without interruption, to the present time, a period of nearly *nine weeks*.

Catarrhal affections continue to prevail. Bronchitis asthenica has been extremely fatal; but the acute form of it, and peripneumonia, have not been frequent in the district (south-west) to which our observations chiefly extend. A case of epilepsy consequent on amenorrhœa, in a strong girl of healthy appearance and florid complexion, was relieved by bleeding from the arm, and will probably be cured by a repetition of the remedy. Paralysis has occurred more frequently, and in younger subjects, than is usual.

